# Supplementary material for: Modeling fibrotic alveolar transitional cells with pluripotent stem cell-derived alveolar organoids
Source: Life Sci Alliance. 2023 May 25;6(8):e202201853. doi: 10.26508/lsa.202201853 (PMC10213712; doi:10.26508/lsa.202201853)
Supplement: Supplementary file 12 [file LSA-2022-01853_Supplemental_Data_1.docx]

**Supplemental Experimental Procedures**

## **Human iPSC maintenance**

iPSC from the previously described line r-iPSC1J (1) were cultured using the Cellartis® DEF-CS™ 500 Culture System (Clontech, Y30010) according to manufacturer’s instructions.

## **Differentiation of human iPSC to iAEC2**

Directed differentiation of iPSC to iAEC2 was performed as previously described (2-4) with minor modifications; briefly, iPSC were seeded at 6 million cells per Matrigel-coated (Corning, 354277) T25 flask in Cellartis® DEF-CS™medium (Clontech, Y30010) supplemented with 10 µM Y-27632 (Calbiochem, 688000). 24 hours later, the cells were differentiated to definitive endoderm (DE) using the commercial STEMdiff™ Definitive Endoderm Kit (Stemcell Technologies, 05110) for 3 days. For the next differentiation steps, cells were cultured in chemically defined media in complete serum-free differentiation medium (cSFDM) (Table S1). Following the DE induction, medium was switched to “DS/SB” medium for anterior foregut endoderm induction (Table S2) for another 3 days. At day 6 of differentiation, the medium was switched to “CBRa” medium (Table S3) until day 15 of differentiation. At day 8 of differentiation, a 1:3 ratio cell split was introduced. For iAEC2 differentiation and maturation, sorted CD47^high^/CD26^low^ lung progenitor cells according to the previously published protocols (2-4) were cultured in 20 µl drops of growth factor-reduced Matrigel (Corning, 354230) in 96-well plates with lung epithelium maturation medium “CK+DCI” (Table S4) and passaged by single cell dissociation every 2-3 weeks as described previously using dispase (Corning, 354235) (2, 3). At passage 2-3, alveolospheres were cryopreserved in freezing medium consisting of 90 % embryonic stem cell (ESC)-qualified fetal bovine serum (FBS) (Thermo Fisher, 16141061) and 10 % dimethyl sulfoxide (DMSO) (Sigma-Aldrich, D8418) according to the previously published protocols (2, 3).

## **Fluorescence-activated cell sorting (FACS)**

At day 15 of differentiation, lung progenitor cells were dissociated, resuspended in FACS buffer containing 1x HBSS (-/-) (Gibco, 14175-053), 2% ESC-qualified FBS, 10 μM Y-27632 (Calbiochem, 688000) and 200 ng/ml Primocin (Invivogen, NC9141851) and stained for FACS analysis as earlier described (4) using primary antibodies or isotype controls as outlined in Table S5. Cell sorting was performed on a Sony SH800 Cell Sorter (Sony Biotechnology) and analysis acquired using the accompanying software (Sony Biotechnology). For intracellular staining of NKX2.1, FACS buffer containing 2% FBS (Thermo Fisher, 10270-106) and 2 mM ethylenediaminetetraacetic acid disodium salt dihydrate (EDTA) (Sigma Aldrich, E4884) in phosphate-buffered saline (PBS) (-/-) (Thermo Fisher, 10010-015) was used and prior to antibody staining, cells were first fixed with Fix Buffer I (BD Biosciences, 557870) and permeabilised with Perm Buffer III (BD Biosciences, 558050). Analysis and plots were generated using the software FlowJo v.10 (BD Biosciences).

## **Reverse transcriptase quantitative PCR (qRT-PCR)**

RNA from alveolospheres was isolated using TRIzol™ Reagent (Invitrogen, 15596026) according to manufacturer’s instructions. mRNA was further purified from the aqueous phase using the RNeasy Micro Kit (Qiagen, 74004) according to manufacturer’s instructions. Reverse transcription was performed using the High-Capacity cDNA Reverse Transcription Kit (Applied Biosystems, 4368813) according to manufacturer’s instructions on a Peltier Thermal Cycler PTC-200 (MJ Research). For qRT-PCR analysis, individual TaqMan® Gene Expression Assays (Applied Biosystems) were used with an amount of 1.3-2 ng cDNA per assay and TaqMan® Low Density Arrays (Applied Biosystems, 4342253) were used with a total amount of 200 ng cDNA per sample (2 ng cDNA per assay) and run for 45 cycles using the QuantStudio7 system (Applied Biosystems). CT values above 40 were considered as no expression. Gene expression was normalised to the appropriate endogenous controls as indicated and expressed as fold changes calculated using the 2^(−ΔΔCT) method relative to the corresponding control samples as denoted for each case. Where indicated, human lung total RNA (Invitrogen, AM7968) was converted to cDNA and used as a reference sample for normalisation. A list of all TaqMan® Gene Expression Assays used in this study is provided in Table S6.

**RNA-sequencing**
RNA from alveolospheres was isolated as already described. Concentration and quality of the RNA was determined using the RNA 6000 Nano Assay on the Agilent Bioanalyzer (Agilent Technologies). Libraries were prepared by Source Bioscience (Nottingham, United Kingdom) using the NEBNext® Ultra™ II Directional RNA Library Prep Kit for Illumina® with mRNA module (New England Biolabs Inc., E7760) and sequenced (50 ng, 150bp paired-end) on a NovaSeq 6000 (Illumina).

## **Bioinformatic analysis of RNA-sequencing data**

The fastq files were processed with the bcbio-nextgen v 1.2.3 pipeline using Hisat2 (v2.2.0) as the aligner and Salmon (v1.1.0) as the pseudo-aligner. The reference genome was hg38, ensembl annotation release 99. In downstream analyses the TPM values generated from Salmon were used as the expression values. The differential expression was performed using the Salmon generated pseudo-counts processed with tximport in DESeq2 with apeglm shrinkage to generate log2 fold changes and multiple testing adjusted *p*-values (*p(adj)*). Principle component analysis (PCA) was performed based on the top 1000 transcripts by coefficient of variation. To generate the list of differentially expressed genes (DEG), dysregulated transcripts were defined by an absolute log2 fold change of 0.7 and a FDR-adjusted *p*-value (*p(adj)) <* 0.05 (List S1). The gene ontology analysis was generated by The Database for Annotation, Visualization and Integrated Discovery (DAVID, v6.8, <https://david.ncifcrf.gov/> , accessed on 27 April 2021)(5, 6) based on the dysregulated transcripts defined by an absolute log2 fold change of 0.7 and *p(adj) <* 0.05 (Lists S2-S9), selected hits defined by *p(adj) <* 0.05 for graphs. The differentially expressed genes in alveolospheres stimulated with the FC were compared to publicly available data from human AEC2 isolated from IPF patients (7), GEO Accession: GSE94555. The gene sets were split into upregulated transcripts and downregulated transcripts and respective intersections were calculated. Venn-diagrams were produced by utilising the following online tool: <http://bioinformatics.psb.ugent.be/webtools/Venn/> and the complete lists of commonly upregulated and downregulated transcripts are presented in Lists S10 and S11, respectively. Heatmaps were generated using Morpheus, (<https://software.broadinstitute.org/morpheus>), linkage method average and one minus Pearson correlation. For heatmaps and expression plots log2(TPM) were used unless otherwise stated.

## **Deconvolution analysis**

Deconvolution of bulk RNA-seq data to infer cell type proportion relative to native tissue was performed using the Bisque package in R v3.5.2 using the single-cell reference-based approach as already described (8, 9) based on a publicly available single-cell RNA-seq dataset (10), GEO Accession: GSE135893. Cells were filtered to only include epithelial cell populations from non-fibrotic donors and IPF patients. Furthermore, deconvolution to infer cell types relative to alveolar organoid differentiation was performed using reference dataset GSE150068 (11) is deposited in the GitHub repository: <https://github.com/Lung-bioengineering-regeneration-lab/FC_alveolospheres>.

## **Western blot analysis**

For intracellular protein retrieval, alveolospheres were dissociated from the Matrigel as described previously (2, 3) and lysed in RIPA Lysis and Extraction Buffer (Thermo Fisher, 89900) supplemented with cOmplete™ Protease Inhibitor Cocktail (Roche, 11697498001) and PhosSTOP™ inhibitors (Roche, 4906837001) on ice for 30 min. The lysate was then obtained by centrifugation at 15 000 x g for 15 min at 4 °C. Human lung tissue was homogenised for 2x 30 seconds at 25 Hz in ice cold PBS with cOmplete™ Protease Inhibitor Cocktail and PhosSTOP™ inhibitors, then lysed by addition of RIPA Lysis and Extraction buffer. The lysate was then obtained by centrifugation at 14 000 x g for 10 min at 4 °C. The total protein concentration was measured with the Pierce™ BCA Protein Assay Kit (Thermo Fisher, 23225). A total of 10-20 µg of intracellular protein was resolved on precast NuPAGE™ Novex™ 12 % Bis-Tris protein gels (Invitrogen, NP0343 and NP0341) and transferred to Invitrolon™ PVDF membranes (Invitrogen, LC2005). Blots were incubated with primary antibodies overnight at 4 °C as specified in Table S5. Species-specific secondary antibodies conjugated to infrared (IR) dyes of either 680 nm or 800 nm wavelengths (LiCOR Biosciences) were used at a dilution of 1:10 000 and the blots were visualised using the Odyssey Imaging System (LiCOR Biosciences), automatic setting of channel intensities, 169 µm resolution and quality-highest. The blots were blotted sequentially with anti-pro-SP-C (Merck, AB3786) with either anti-GAPDH (Abcam, ab9482) or anti-β-actin (Abcam, ab8229) and visualised, then re-blocked with blocking buffer containing TBS + 0.1 % Tween-20 (Takara, T9142) + 5 % BSA (Sigma, A6003) and re-incubated with anti-SP-B (Abcam, ab40876) overnight and visualised with secondary antibodies as described. The signals were quantified using the software Image Studio v4.0 (LiCOR Biosciences) on the original blots. For figures, brightness and contrast for the whole blot was adjusted for each channel individually, and regions of interest were cropped from the original blots for figure display.

## **Organoid morphology measurements**

Alveolospheres were imaged for organoid area and count analysis by phase contrast microscopy using the 4x objective with automatic focus on the Incucyte S3 (Sartorius). These images were used for the organoid area and count analysis performed using the Incucyte 2021A software (Sartorius), where organoids with an area of minimum 3000 μm^2^ in size were included. Average organoid areas per well and average counts per batch were used for further analysis. Images in which no organoids were detected were excluded from the analysis. Brightfield images used for quantification of “dense” and “normal” organoids after CC and FC stimulation were taken using the Plan Fluor 4X PhL DL objective on the Eclipse TE2000-U microscope (Nikon) at a resolution of 2560 px × 2160 px, 12-bit format. Organoids were defined as “dense” if they lacked a visible lumen and appeared dark grey in colour, and defined as “normal” if they had a visible lumen. The percentages of each morphological type of organoids are based on blinded, manual counting of the “dense” and “normal” organoids per well.

## **Immunofluorescence microscopy**

Alveolospheres were fixed with 4 % paraformaldehyde for 1 h on ice. Alveolospheres were then permeabilised in blocking buffer containing 5 % normal goat serum (Vector Laboratories, S-1000) with 0.5 % Triton X-100 (Sigma) overnight at 4°C. The primary antibodies were diluted in blocking buffer and incubated at 4 °C overnight as specified in Table S5. Alveolospheres were then washed in blocking buffer and counter-stained with Hoechst33342 at 3.3 μg/ml (ThermoFisher, H3570) and Alexa Fluor 594 phalloidin (Thermo Fisher, A12381), and the following secondary antibodies: goat anti-rabbit IgG Alexa Fluor 488 (Thermo Fisher, A11034, dilution 1:500), goat anti-mouse Alexa Fluor 594 (Thermo Fisher, A21125, dilution 1:500) and goat anti-mouse Alexa Fluor 647 (Thermo Fisher, A21241, dilution 1:500) in blocking buffer at 4 °C overnight. Visualisation was performed by using a Zeiss LSM 880 laser scanning confocal microscope (Zeiss) at a resolution of 1024 px × 1024 px, 16-bit format, pinhole set to 1 airy unit using the Plan-Apochromat 20x/0.8 M27 objective and image processing was performed using the software ZEN v2.3 (Zeiss). The imaging settings were: for pro-SP-C/E-cadherin/Hoechst, laser strength: 633-10.0%, 488-1.0%, 405-1.0%, master gain: 800 (633 and 405) and 750 (488), digital gain: 633-3.0, 488-0.5, 405-1.0; for vimentin/E-cadherin/Hoechst, laser strength: 633-10.0%, 488-1.0%, 405-1.0%, master gain: 800 (all lasers), digital gain: 633-3.0; 488-10.0; 405-1.0; for keratin 17/keratin8/Hoechst, laser strength: 594-10.0%; 488-1.0%; 405-1.0%, master gain: 800 (all lasers), digital gain: 594-10.0; 488-2.5; 405-1.0; for keratin 17/keratin5/Hoechst, laser strength: 594-10.0%; 488-1.0%; 405-1.0%, master gain: 800 (all lasers), digital gain: 594-1.0; 488-2.5; 405-1.0; for pro-SP-C/keratin5/Hoechst, laser strength: 594-10.0%; 488-1.0%; 405-1.0%, master gain: 800 (594 and 405) and 750 (488), digital gain: 594-1.0, 488-0.5, 405-1.0; for collagen type 1/E-cadherin/Hoechst, laser strength: 633-10.0%; 488-1.0%; 405-1.0%, master gain: 800 (all lasers), digital gain: 633-3.0; 488-1.5; 405-1.0; for collagen type 1/Hoechst, laser strength: 488-1.0%; 405-1.0%, master gain: 800 (all lasers), digital gain: 488-1.5; 405-1.0; for SP-B/Phalloidin/Hoechst, laser strength: 594-2.0%; 488-2.0%; 405-2.0%, master gain: 594-620; 488-569; 405-650, digital gain: 594-1.0; 488-1.0; 405-1.0. The images shown in the figures are maximum intensity projections of 10 vertical z-stack images (size 6.173 µm (6.175 µm for keratin 17/8)). For Fig S1E, single plane images were captured at 512 px × 512 px, 8-bit format using the LD Plan-Neofluar 20x/0.4 Korr M27 objective. For negative control staining of pro-SP-C, normal human lung fibroblasts (Lonza, CC-2512, donor: 0000608197) were embedded in Matrigel (7500 cells/µl), stained for pro-SP-C and Hoechst in parallell with alveolospheres as described and imaged using a LD Plan-Neofluar 20x/0.4 Korr M27 objective. For positive control staining of KRT5, normal human bronchial epithelial cells (Lonza, CC-2540, donor: 28359) were embedded in Matrigel (1500 cells/µl), stained for KRT5 and Hoechst in parallell with alveolospheres as described and imaged using a LD Plan-Neofluar 20x/0.4 Korr M27 objective. For images in figures, brightness and contrast were adjusted for optimal signal display of each fluorophore and applied to the entire image, and were kept consistent within each staining combination and matched with no primary antibody controls. Display settings are: for E-cadherin, black: 15000 (min 0), gamma 1.00, white: 65535 (max 65535); for pro-SP-C, black: 3000 (min 0), gamma: 1.00, white: 25000 (max 65535); for vimentin, black: 30000 (min 0), gamma: 1.00, white: 65535 (max 65535), for keratin 17, black: 15000 (min 0), gamma: 1.00, white: 65535 (max 65535); for keratin 8, black: 18400 (min 0), gamma: 1.00, white: 65535 (max 65535); for keratin 5, black: 5000 (min 0), gamma: 1.00, white: 65535 (max 65535); for collagen type 1 (permeabilised), black: 25000 (min 0), gamma: 1.00, white: 65535 (max 65535); for collagen type 1 (non-permeabilised), black: 11455 (min 0), gamma: 1.00, white: 45010 (max 65535); for SP-B, black: 6 (min 0), gamma: 1.00, white: 255 (max 255); for phalloidin, black: 0 (min 0), gamma: 1.00, white: 150 (max 255).

For determination of pro-SP-C or KRT5 expression, alveolospheres were imaged in a CV7000S microscope (Yokogawa) using the 10x UPL SAPO objective with the laser strength: 405 nm-25%, 488 nm-25%, 561 nm-30%. For the 405 channel capturing DNA, exposure time was 300 ms, binning 2x2, for 488 channel capturing pro-SP-C, exposure time was 200 ms and binning 2x2, for 561 channel capturing KRT5, exposure time was 150 ms and binning 2x2. Five images per each 96-well were taken to capture the majority of organoids, and maximum intensity projections were generated from 4 vertical z-stacks of 10 µm/stack. Alveolospheres were manually classified as either pro-SP-C positive or negative and KRT5 positive or negative based on visual assessment of the staining, then counted and expressed as percentage proportions of total organoid count.

For quantification of KRT17 and KRT5 expression, alveolospheres were imaged in a CV7000S microscope (Yokogawa) using the 4x UPL SAPO objective with the laser strength: 405 nm-25%, 488 nm-25%, 561 nm-30%. For the 405 channel capturing DNA, exposure time was 300 ms, binning 2x2, for 488 channel capturing KRT17, exposure time was 200 ms and binning 2x2, for 561 channel capturing KRT5, exposure time was 150 ms and binning 2x2. Two images per each 96-well were taken to capture the majority of organoids, and maximum intensity projections were generated from 30 vertical z-stacks of 10 µm/stack. Images were processed using the CellVoyager software (Yokogawa). Quantification of fluorescent area over Hoechst was performed using HALO v.3.4.2986.230 (Indica Labs). Images were classified using the Random Forest classifier type by defining multiple image areas as either organoid, background or Matrigel. The analysis setting Area Quantification FL v.2.2.2 was used to obtain the fluorescent areas of KRT17 (min intensity 115), KRT5 (min intensity 38.3) and Hoechst (min intensity 76.6) in the organoids (background and Matrigel excluded from analysis).

For determination of KRT17 expression relative to organoid morphology, organoids immunostained for KRT17 were first manually scored as “high” if they appeared fully stained, “low” if they appeared partially stained, and “negative” if no visible staining was detected. Separately, organoid morphology was classified in brightfield images corresponding to the same areas assessed for KRT17 staining. The KRT17 staining score of each organoid was linked with the organoid morphology type. The image information about stimulation and batch was kept blinded until the final stage of analysis.

**Enzyme-linked immunosorbent assay (ELISA)**

Secreted protein levels were assessed in conditioned medium from alveolospheres by ELISA for human fibronectin (Thermo Fisher, BMS2028), human tenascin (Abcam, ab213831) and human pro-collagen 1α1 (R&D Systems, DY6220-05) according to manufacturer’s instructions.

## **LDH analysis**

LDH analysis was performed using the CyQUANT™ LDH Cytotoxicity Assay Kit (Invitrogen, C20301) using fresh cell culture medium according to manufacturer’s instructions.

**Supplemental References**

1. Sjogren AK, Liljevald M, Glinghammar B, Sagemark J, Li XQ, Jonebring A, Cotgreave I, Brolén G, Andersson TB. Critical differences in toxicity mechanisms in induced pluripotent stem cell-derived hepatocytes, hepatic cell lines and primary hepatocytes. *Arch Toxicol* 2014; 88: 1427-1437.

2. Jacob A, Morley M, Hawkins F, McCauley KB, Jean JC, Heins H, Na C-L, Weaver TE, Vedaie M, Hurley K, et al. Differentiation of Human Pluripotent Stem Cells into Functional Lung Alveolar Epithelial Cells. *Cell Stem cell* 2017; 21: 472-488.e410.

3. Jacob A, Vedaie M, Roberts DA, Thomas DC, Villacorta-Martin C, Alysandratos K-D, Hawkins F, Kotton DN. Derivation of self-renewing lung alveolar epithelial type II cells from human pluripotent stem cells. *Nat Protoc* 2019; 14: 3303-3332.

4. Hawkins F, Kramer P, Jacob A, Driver I, Thomas DC, McCauley KB, Skvir N, Crane AM, Kurmann AA, Hollenberg AN, et al. Prospective isolation of NKX2-1-expressing human lung progenitors derived from pluripotent stem cells. *J Clin Invest* 2017; 127: 2277-2294.

5. Sherman BT, Hao M, Qiu J, Jiao X, Baseler MW, Lane HC, Imamichi T, Chang W. DAVID: a web server for functional enrichment analysis and functional annotation of gene lists (2021 update). *Nucleic Acids Res* 2022; 50: W216-221.

6. Huang da W, Sherman BT, Lempicki RA. Systematic and integrative analysis of large gene lists using DAVID bioinformatics resources. *Nat Protoc* 2009; 4: 44-57.

7. Xu Y, Mizuno T, Sridharan A, Du Y, Guo M, Tang J, Wikenheiser-Brokamp KA, Perl A-KT, Funari VA, Gokey JJ, et al. Single-cell RNA sequencing identifies diverse roles of epithelial cells in idiopathic pulmonary fibrosis. *JCI Insight* 2016; 1: e90558-e90558.

8. Stegmayr J, Alsafadi HN, Langwinski W, Niroomand A, Lindstedt S, Leigh ND, Wagner DE. Isolation of high yield and quality RNA from human precision-cut lung slices for RNA-sequencing and computational integration with larger patient cohorts. *Am J Physiol Lung Cell Mol Physiol* 2020; 320(2): L232-L240.

9. Jew B, Alvarez M, Rahmani E, Miao Z, Ko A, Garske KM, Sul JH, Pietiläinen KH, Pajukanta P, Halperin E. Accurate estimation of cell composition in bulk expression through robust integration of single-cell information. *Nat Commun* 2020; 11: 1971.

10. Habermann AC, Gutierrez AJ, Bui LT, Yahn SL, Winters NI, Calvi CL, Peter L, Chung M-I, Taylor CJ, Jetter C, et al. Single-cell RNA sequencing reveals profibrotic roles of distinct epithelial and mesenchymal lineages in pulmonary fibrosis. *Sci Adv* 2020; 6: eaba1972.

11. Kathiriya JJ, Wang C, Zhou M, Brumwell A, Cassandras M, Le Saux CJ, Cohen M, Alysandratos KD, Wang B, Wolters P, et al. Human alveolar type 2 epithelium transdifferentiates into metaplastic KRT5(+) basal cells. *Nat Cell Biol* 2022; 24: 10-23.
